# Cognitive effects of STN-DBS on mental rotation performance in Parkinson’s disease

**DOI:** 10.1038/s41598-026-52880-2

**Published:** 2026-05-19

**Authors:** Marleen J. Schoenfeld, Alessandro Gulberti, Nicole David, Wolfgang Hamel, Monika Pötter-Nerger, Andreas K. Engel, Christian K. E. Moll

**Affiliations:** 1 Department of Neurophysiology and Pathophysiology, University Medical Center Hamburg-Eppendorf, Martinistr. 52, 20246 Hamburg, Germany; 2 Hamburg Center of Neural and Cognitive Systems (HCNS), University Medical Center Hamburg-Eppendorf, Martinistr. 52, 20246 Hamburg, Germany; 3 Department of Neurology, University Medical Center Hamburg-Eppendorf, Martinistr. 52, 20246 Hamburg, Germany; 4 Department of Neurosurgery, University Medical Center Hamburg-Eppendorf, Martinistr. 52, 20246 Hamburg, Germany

**Keywords:** Parkinson’s disease, Deep brain stimulation, Subthalamic nucleus, Cognitive symptoms, Mental rotation, Diseases, Neurology, Neuroscience

## Abstract

In Parkinson’s disease (PD), cognitive symptoms progressively worsen with disease progression and present a major clinical challenge that remains difficult to treat. Deep brain stimulation of the subthalamic nucleus (STN-DBS) is a widely used and clinically validated therapy for motor symptoms in PD; however, its cognitive effects remain underexplored. In this study, we examined how STN-DBS influences executive functioning, focusing on a well-established mental rotation task. In the task, patients were shown two images (reference, comparison) and were asked to decide whether the comparison image was rotated or mirrored. Twelve PD patients (6 women; mean age: 60 ± 10 years; mean disease duration: 12 ± 6 years) completed the task under two conditions: with therapeutic STN-DBS turned on and turned off. Behavioural cognitive performance was correlated with the volume of tissue activated, as determined by MR based electrode reconstruction. Results indicated that STN-DBS significantly improved mental rotation accuracy, suggesting a beneficial effect on visuospatial executive processing. Notably, stimulation involving limbic-associated regions of the right STN was associated with impaired performance, highlighting the relevance of stimulation site and its functional connectivity. Together, these findings contribute to the growing body of evidence that STN-DBS produces region-specific effects on distinct cognitive domains.

## Introduction

Cognitive symptoms in Parkinson’s disease (PD) become increasingly prevalent and severe with disease progression and substantially impact patients’ quality of life^1–3^. Cognitive impairments span multiple domains, including executive functioning^4–6^, cognitive flexibility^7^ and visuospatial functioning^8–10^.

Current PD treatment primarily targets motor symptoms and typically includes standard dopaminergic medication and deep brain stimulation of the subthalamic nucleus (STN-DBS). While the motor benefits of STN-DBS are widely recognised^11^, DBS has also been shown to influence cognitive processes^12–15^. However, the reported effects on cognitive domains remain inconsistent^16,17^. It is therefore crucial to further investigate how and under what conditions STN-DBS modulates cognitive performance in PD.

An important factor contributing to the effect variability is the precise electrode placement within the STN. While dorsal STN placement of the DBS electrode is often associated with more favourable outcomes^18^, ventral STN placement has been linked to cognitive decline^15,19,20^, and limbic STN placement with other detrimental effects, including the emergence of manic symptoms^21^. In addition, some evidence suggests that STN-DBS induces lateralized effects on specific cognitive domains^22^ with stimulation of the left STN primarily affecting verbal abilities, and stimulation of the right STN influencing other cognitive domains^14,22,23^. This further highlights the importance of considering the positioning of electrodes when investigating the cognitive effects of STN-DBS.

Visuospatial functioning represents a cognitive domain of particular importance to everyday activities such as driving, cooking, shopping, and social interactions, as it supports navigation, object manipulation, and interpretation of spatial relationships^24^. Mental rotation is a key aspect of visuospatial functioning and assesses the ability to mentally manipulate and compare visual stimuli. It has been investigated using a range of different psychometric paradigms, including adaptations of the classic mental rotation task by Shepard & Metzler (1971) and Vandenberg & Kuse (1978). In this task, participants judge whether two visual patterns are identical or mirror images. Previous studies reported that PD patients are impaired in performing the mental rotation task, revealing significantly increased error rates compared to healthy controls^8–10,27^, independent of dopamine medication^28^. Others argue that mental rotation stays relatively intact in PD^29–31^. Notably, although these studies assessed the influence of dopaminergic medication on mental rotation, they did not explore the effects of STN-DBS activation state nor their electrode placement. Leveraging the opportunity to modulate STN activity by switching DBS on or off during performance of the mental rotation task offers a unique opportunity to more specifically address the short-term and within-subject cognitive effects of STN-DBS.

In this study, we test the effects of DBS on cognitive function using the mental rotation paradigm adapted from Shepard & Metzler (1971). To this end, patients performed a mental rotation task under bilateral STN-DBS ON (ON-DBS) and OFF (OFF-DBS) conditions. We hypothesized a better mental rotation performance in ON-DBS compared to OFF-DBS, based on previous reports of cognitive improvements with DBS switched on versus off^12,13,23,32^. Further, we anticipated improved performance in mental rotation to correlate with motor symptom relief. In addition, we expected electrode placement to influence performance, with ventral (limbic-associated) stimulation correlating with worsened task performance, and more dorsally localized stimulation correlating with improved performance.

## Methods

### Participants

Twelve individuals with advanced PD (6 females, mean age: 60 ± 10.3 years; mean disease duration: 11.8 ± 5.5 years, Hoehn & Yahr^33^ stage: 2.9 ± 0.8 (H&Y)) gave written informed consent to participate in the study. The study was conducted in accordance with the Declaration of Helsinki and was approved by the Ethics Committee of the Medical Association Hamburg, Germany (approval number PV4298; Ethik-Kommission der Ärztekammer Hamburg). All patients had normal hearing and normal or corrected-to-normal vision. All patients had previously undergone bilateral implantation of DBS electrodes targeting the STN (mean time since implantation: 31.2 ± 24.8 months, range 3–68 months, see Table 1). Following implantation, their levodopa-equivalent daily dose (LEDD) was 512 ± 140 mg, calculated using standard conversion factors^34^ (see Table 1 for individual values). Medication and stimulation parameters, including bipolar or multi-contact configurations, were determined during routine postoperative programming at the treating neurological outpatient clinic and were not modified for the purposes of the experiment, ensuring that patients were tested under their clinically optimized therapeutic settings.

**Table 1:** Clinical and demographic characteristics.

Case Gender Age	Disease duration (years)	Pre-op H&Y	Pre-op LEDD	Pre-op UPDRS		DBS parameters for: Left electrode (1st row) Right electrode (2nd row)	Months since DBS Implantation	Post-op H&Y	Post-op LEDD	Post-op UPDRS				Session order: 1= ON-DBS 1^**st**^ 2= OFF-DBS 1^st^
				DOPA-OFF	DOPA-ON					DOPA-OFF OFF-DBS	DOPA-OFF ON-DBS	DOPA-ON ON-DBS	DOPA-ON OFF-DBS	
1, M, 42	9	4	725 mg	40	24	180 Hz, 1-, 2-, 2.1 V, 60 μs 180 Hz, 9-, 10-, 2.0 V, 60 μs	33	2	475 mg	20	8	7	11	2
2, M, 68	6	N/A	N/A	38	8	130 Hz, 2-, 3-, 1.7 V, 60 μs 130 Hz, 10-, 11-, 2.0 V, 60 μs	3	2	757 mg	30	15	5	12	1
3, F, 71	8	N/A	1010 mg	N/A	N/A	170 Hz, 2-, 3-, 1.8 V, 60 μs 170 Hz, 10-, 11-, 2.1 V, 60 μs	3	2	300 mg	40	11	4	30	1
4, M, 53	14	2	1217 mg	30	19	130 Hz, 0-, 1-, 3 + , 3.0 V, 60 μs 130 Hz, 9-, 10-, 11 + , 2.0 V, 60 μs	52	N/A	N/A	N/A	N/A	N/A	N/A	2
5, F, 71	15	3	683 mg	48	27	130 Hz, 1-, 3 + , 2.5 V, 60 μs 130 Hz, 5-, 7 + , 2.5 V, 60 μs	68	N/A	662 mg	44	N/A	19	25	2
6, M, 70	25	4	939 mg	51	27	130 Hz, 2-, 3-, 2.5 V, 60 μs 130 Hz, 10-, 11-, 3.0 V, 60 μs	30	N/A	531 mg	N/A	N/A	22	N/A	1
7, M, 49	11	3	613 mg	25	17	130 Hz, 1-, 2-, 3.1 V, 60 μs 130 Hz, 9-, 10-, 3.0 V, 60 μs	54	3	550 mg	N/A	N/A	21	N/A	1
8, F, 58	15	2	1500 mg	23	10	210 Hz, 9-,10-, 2.8 V, 60 μs 210 Hz, 1-,2-, 2.3 V, 60 μs	36	1.5	613 mg	N/A	N/A	7	N/A	1
9, M, 48	6	2	1085 mg	31	3	125 Hz, 2-, 3.2 V, 3-, 3.5 V, 60 μs 125 Hz, 9-, 3.0 V, 60 μs	5	N/A	403 mg	N/A	N/A	2	N/A	2
10, F, 57	10	N/A	1285 mg	36	9	130 Hz, 1-, 2-, 2.5 V, 60 μs 130 Hz, 9-, 10-, 2.8 V, 60 μs	16	N/A	350 mg	N/A	N/A	N/A	N/A	1
11, F, 65	16	3	350 mg	26	12	160 Hz, 1-, 2-, 2.0 V, 60 μs 160 Hz, 10-, 2.0 V, 60 μs	68	N/A	438 mg	41	22	15	N/A	2
12, F, 69	7	3	433 mg	29	4	180 Hz, 1-, 2-, 1.2 V, 60 μs 180 Hz, 9-,10-, 2.3 V, 60 μs	7	N/A	630 mg	N/A	N/A	N/A	N/A	2

In column “DBS parameters” values reported are stimulation frequency in Hz, active contacts, pulse amplitude in volts and pulse width in μs for the left and right electrode, respectively. For the left electrode, contact 0 was the most ventral and contact 3 was the most dorsal. For the right electrode, contact 4 (or 8 in case of Activa PC stimulator) was the most ventral and contact 7 (or 11 in case of Activa PC stimulator) was the most dorsal. The N/Avalues were missing randomly and not due to specific clinical characteristics but rather attributable to clinical feasibility and logistical and practical factors. Abbreviations: N/A = not available. H&Y = Hoehn & Yahr scale. Op = operation for DBS electrodes implantation. UPDRS-III = Unified Parkinson’s Disease Rating Scale, motor-subscore (part III). LEDD = levodopa equivalent daily doses. Session 1 corresponds to ON-DBS condition first followed by OFF-DBS, and session 2 corresponds to OFF-DBS first followed by ON-DBS.

To examine patients’ disease progression and severity, patients’ motor abilities were assessed with the motor-subsection (Part III) of the Unified Parkinson’s Disease Rating Scale^35^ (UPDRS) and the H&Y scale prior to the study. We extracted UPDRS-III and H&Y scores as well as LEDD from patients’ clinical records, using values closest in time to the study visit. Stimulation parameters (e.g., voltage, frequency, pulse width, active contacts) were documented on the day of participation (see Table 1).

### Experimental design

The experiment was completed in two sessions on the same day: one session was conducted under the ON-DBS condition, i.e., during the conventional bilateral therapeutic STN-DBS, and one session under the OFF-DBS condition, i.e., with the DBS device switched off. The order of session was counterbalanced across patients. Thus, patients either performed the task under the ON-DBS condition first followed by the OFF-DBS condition (session 1), or under the OFF-DBS condition first followed by the ON-DBS condition (session 2), see Table 1. Throughout the experiment, all patients were on their regular medication. Because dopaminergic treatment can affect executive and visuospatial performance, both sessions were conducted under the same medication conditions. This helped minimise confounding factors when comparing stimulation effects and also ensured patients’ physical comfort during the experiment.

For each patient, stimulation contacts, amplitude and pulse duration were the same as for therapeutic high-frequency stimulation (see Table 1). A minimum washout period of 40 min elapsed between switching off the DBS device and performing the task in the OFF-DBS session (mean time: 71.2 ± 21.5 min, range 40–115 min). Based on previous studies, this period of time has been shown to be long enough to induce significant worsening of motor symptoms^36,37^ as well as cognitive symptoms^13,14,23^, and was therefore deemed sufficient for revealing potential cognitive effects of stimulation withdrawal for this paradigm. To minimize potential carryover effects from task repetition, the interval between sessions (ON-DBS, OFF-DBS) was at least 66 min (mean time: 103.6 ± 59.6 min, range 66–283 min).

**Fig. 1:**
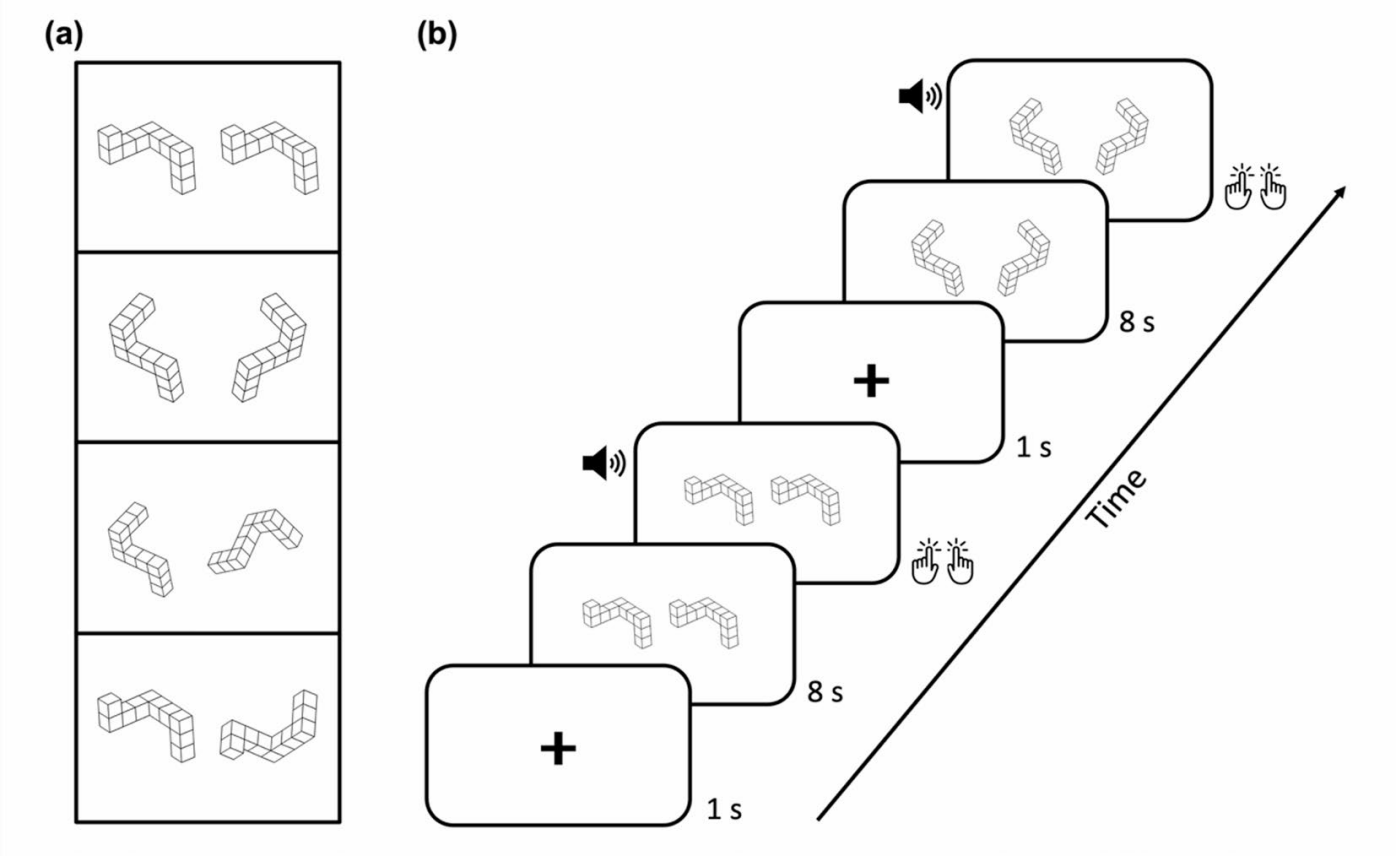
Experimental design and stimuli. (**a**) Exemplary stimuli used in the mental rotation task adapted from Shepard & Metzler’s 3D mental rotation task. From top to bottom, the stimuli are identical 0°, mirrored 0°, identical 90° and mirrored 180°. (**b**) Experimental design for the mental rotation task. A fixation cross was presented for 1 s, followed by the simultaneous display of two stimuli for 8 s. After 8 s, patients were asked to judge whether the two stimuli were ‘identical’ or ‘mirrored’ by pressing the corresponding button on the computer keyboard. An auditory sound indicated to participants to respond. The next trial was displayed immediately.

In each session, participants were comfortably seated in front of a computer screen to perform a computer-based version of the mental rotation task adapted from Shepard & Metzler (1971). The task entailed stimuli as 3D objects, i.e., ten solid cubes connected face-to-face to form rigid, armlike structures with precisely three right-angled “elbows”^25^. Stimuli were presented in four different orientations angled in the picture plane: 0° (upright), 90° (clockwise), 180° (upside down), and 270° (90° counterclockwise) either as an identical pair or as a mirrored version of its counterpart (Fig. 1a).

Each trial of the task began with the presentation of a fixation cross for 1 s, followed by a simultaneous display of two 3D stimuli for 8 s (Fig. 1b). After the interval, participants were asked to judge whether the stimuli were ‘identical’ or ‘mirrored’ by pressing the corresponding key on the keyboard: ‘N’ for identical (left finger) and ‘M’ for mirrored (right finger). Participants were instructed to keep their fingers on the response keys and to respond as quickly and accurately as possible after hearing an acoustic ‘Go’ signal, indicating the end of the 8-s interval and the onset of the response phase. A fixed presentation window of 8 s was used to standardize response times and to control for potential differences in motor response latency between ON-DBS and OFF-DBS sessions, as PD patients have been shown to exhibit slower responses during stimulation-off conditions^10^. Task compliance and response behaviour were visually monitored by the experimenter throughout the session. After participants responded, the next trial was displayed immediately, starting with the fixation cross. In total, the task entailed 128 trials for each of the experimental conditions.

Stimuli were presented and responses were collected using a computerized version of the mental rotation task originally hosted at http://toblerserv.unibe.ch/~devil/rotation/ (University of Bern, Institute of Psychology, Bern, Switzerland; site no longer active). The task was run on a MacBook Pro operating with OS X Yosemite (10.10).

Task performance was assessed using error rate (%) as the dependent variable, calculated as the average percentage of incorrect responses across all orientations and planes. To evaluate the effect of stimulation, we computed the difference in error rate between the OFF-DBS and ON-DBS conditions (OFF-DBS minus ON-DBS) for each participant. Additionally, response times (RTs) in milliseconds were recorded to verify compliance with task instructions and to monitor potential differences in response behaviour.

### Statistical analysis

To investigate changes in the task, error rates (%) between the four different orientations of the mental rotation task were compared. If not stated otherwise frequentist statistics were used, and data were analysed using repeated measures analysis of variance (ANOVA). In case of significant effects, post hoc t-tests were conducted with Bonferroni correction for multiple comparisons. Correlations were examined using Pearson’s correlation. All reported p-values for t-tests were two-tailed, and the significance level was set at *p* < 0.05. To test whether there were no differences in response times across items and stimulation sessions, Bayesian statistics were employed. Bayesian statistics are particularly well suited to assess if data favoured the null hypothesis compared to the alternative hypothesis^38^. To this end, we conducted the same analysis as with frequentist statistics^39,40^ using default priors and report Bayes factor exclusion (BF_exclusion_) for the interaction between stimulation session and items. Data were analysed using R^41^ (version: 4.3.1, RRID: SCR_001905) and RStudio^42^ (version 2023.06.1 + 524, RRID: SCR_000432). Statistical analyses were performed using the opensource software JASP^43^ (version 0.19.3, RRID: SCR_015823).

### Electrodes localization and stimulation modelling

DBS leads were localized using the default parameters of the Lead-DBS toolbox^44^ (www.lead-dbs.org; version 2.5.3; RRID:SCR_002915), and following the processing pipe-line as described^45^. Briefly, preoperatively acquired high-resolution T1 weighted MRIs were linearly co-registered with post-operative control CT scans using a two-stage linear registration (rigid followed by affine) as implemented in the Advanced Normalization Tools^46^ (ANTs; RRID:SCR_004757; http://stnava.github.io/ANTs/) or using the registration method ‘FLIRT’ as implemented in FSL, if results of ANTs co-registration were unsatisfactory^47,48^ (RRID:SCR_002823). In addition, T2 weighted MRIs were linearly co-registered to T1 MRIs using SPM12^49^ (RRID:SCR_007037; http://www.fil.ion.ucl.ac.uk/spm/software/). The images were then nonlinearly normalized into standard space (ICBM 2009b NLIN, Asym) using advanced normalization tools based on the preoperative MRIs and the ‘effective (low variance)’ strategy as implemented in Lead-DBS. This procedure used preoperative MRIs (T1 and T2) and registered the STN to a template atlas^50^. Electrode localization involved a combination of automated and manual steps. Lead-DBS functions were first used for automated refinement, including brain shift correction and alignment with anatomical landmarks. Electrode trajectories were then manually defined by selecting the electrode tip and a second point along the trajectory in multi-planar views. Localization accuracy was verified by visual inspection of co-registration quality and anatomical plausibility across imaging modalities. The Medtronic 3389 lead was selected for all patients.

#### Stimulation modelling

The volume of tissue activated (VTA) was calculated using a finite element method based on clinically optimized stimulation parameters^44,51^, while the spread of the electric fields was estimated for homogenous tissue following the indications reported in Horn et al. (2017) and in Petry-Schmelzer et al. (2019), with a conductivity of $\sigma$ = 0.1 S/m and a VTA-threshold set at an electrical isolevel field of 0.19 V/mm. VTAs were calculated for the active contacts on each lead with amplitudes ranging from 1.7 to 3 V and a pulse width of 60 μs. Stimulation frequency was not included in the modelling, as the Lead-DBS framework estimates spatial electric field distributions based on amplitude, pulse width, and contact configuration, and has been shown to have minimal impact on VTA estimation^53,54^.

#### Intersection with local structures

For the group analysis, the bilateral intersections between each patient’s VTA and the STN sub-regions (motor, associative, and limbic) defined by the DISTAL atlas^55^ were calculated. These intersections were then correlated with the difference in error rate between the OFF-DBS and ON-DBS conditions (OFF-DBS minus ON-DBS) using the Lead-DBS Group toolbox. Spearman’s correlations were performed between the OFF-DBS vs. ON-DBS error rate difference and the intersection volumes of the VTAs with the local STN sub-regions, separately for bilateral, left, and right hemispheres. A Bonferroni-corrected alpha level of 0.005 was applied to account for the nine computed correlations. Given the exploratory nature of this pilot study, no further corrections for multiple comparisons were implemented at this stage^56^.

#### Sweet and sour spot analysis and predictive outcome

Following established methodologies^57,58^, VTA-based probabilistic stimulation maps were generated in MNI space to identify regions where stimulation was associated with above-mean (“sweet spots”) or below-mean (“sour spots”) error rates. Each patient’s VTA was thresholded at 0.2 V/mm, and analyses were restricted to voxels covered by at least 20% of VTAs. Voxelwise two-tailed Wilcoxon signed-rank tests were then performed to generate a p-map, assessing whether stimulation at each voxel was associated with a significant deviation in error rate from the mean. A correction for VTA size was applied. Finally, the validity of the generated model was assessed using 1000 permutations ("leave-nothing-out") and tenfold randomized cross-validation. Given the small sample size, cross-validation analyses were considered exploratory. In addition, leave-one-out cross-validation (LOOCV) was performed as a sensitivity analysis.

## Results

### DBS improved performance in the mental rotation task

To assess the effect of DBS on task performance, a 4 × 2 × 2 ANOVA was conducted with the within-subject factors *rotation* (0°, 90°, 180°, 270°), *view* (mirrored, identical), and *stimulation* (ON-DBS, OFF-DBS), and error rate (%) as the dependent variable.

**Fig. 2:**
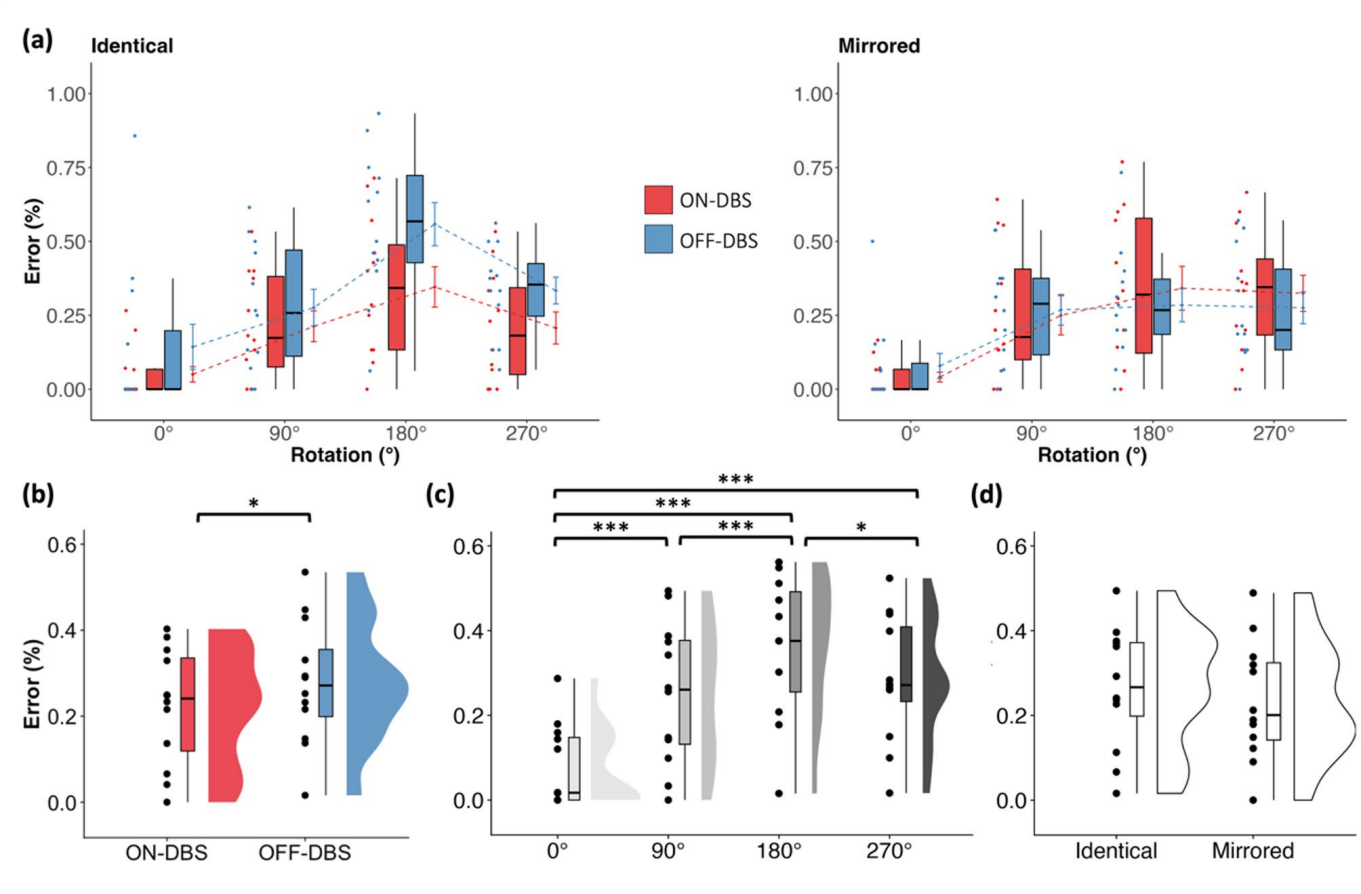
DBS reduced error rates during stimulation (N = 12). (**a**) Comparison of error rates for identical (left) and mirrored (right) items across the four rotation angles (0°, 90°, 180°, 270°) under ON-DBS and OFF-DBS conditions. A significant three-way interaction (stimulation × view × rotation) indicated that DBS improved performance primarily for identical items, particularly at 180°, while DBS was turned on (significant interaction between stimulation x view x rotation). Mean and standard error across subjects are shown, connected with dashed lines, along with individual subject data points and the corresponding boxplots. (**b**) Comparison of error rates with (ON-DBS) and without (OFF-DBS) stimulation suggested that DBS improved performance (significant main effect of DBS). Error rates were averaged over view (identical, mirrored) and rotation angles (0°, 90°, 180°, 270°). Data are shown at the single subject level, as boxplots and distribution. (**c**) Comparison of error rates in the four rotation angles, averaged across stimulation (ON-DBS, OFF-DBS) and view (identical, mirrored). A significant main effect of rotation was found, with the lowest error rate at 0°, followed by 90° and 270° (no difference between them), and the highest error rate at 180°. Data are shown at the single subject level, as boxplots and distributions. (**d**) Comparison of error rates for identical and mirrored items, averaged across all rotation angles (0°, 90°, 180°, 270°) and stimulation (ON-DBS, OFF-DBS). No significant main effect of view was observed, indicating comparable performance. Data are shown at the single subject level, as boxplots and distributions. *p < 0.05, ***p < 0.001.

The analysis revealed a significant main effect of *stimulation* (F_1,11_ = 5.869, *p* = 0.034, η^2^_p_ = 0.348, Fig. 2b) indicating that DBS reduced error rates and thus improved performance. Further, a significant main effect of *rotation* was observed (F_3,33_ = 30.635, *p* < 0.001, η^2^_p_ = 0.736, Fig. 2c), revealing that performance differed across rotation angles. Follow-up t-tests were significant for all rotation angles but one (90° vs 270°, see Table 2) indicating that error rates were smallest in 0° followed equally by 90° and 270°, and largest in 180°. The main effect of *view* was not significant (F_1,11_ = 1.5, *p* = 0.246, η^2^_p_ = 0.12, Fig. 2d) suggesting that task performance did not differ between mirrored and identical item pairs. Importantly, we found a significant interaction between *rotation*, *view* and *stimulation* (F_3,33_ = 3.196, *p* = 0.036, η^2^_p_ = 0.225, Fig. 2a). This suggested that DBS improved performance differently for rotation angles and had the biggest improvement in the identical items with the largest error rate (180°). All other effects were not significant (all *p*-values > 0.05).

**Table 2:** Post hoc tests of 4 × 2 × 2 ANOVA with the within-subject factor rotation.

Rotation		Mean difference	SE	t	Cohen’s d	p_bonf_
0°	90°	− 0.173	0.032	− 5.352	− 0.874	< 0.001
	180°	− 0.304	0.032	− 9.386	− 1.533	< 0.001
	270°	− 0.207	0.032	− 6.379	− 1.042	< 0.001
	270°	− 0.033	0.032	− 1.027	− 0.168	1.000
180°	270°	0.097	0.032	3.007	0.491	0.030

Cohen’s d does not correct for multiple comparisons.

*P*-value adjusted for comparing a family of 6.

Results are averaged over the levels of stimulation, and view.

### Performance in mental rotation task did not correlate with motor function

We hypothesized that improvements in mental rotation performance with *ON-DBS* would be associated with improvements in motor symptoms, i.e., patients who showed greater motor benefit from DBS would also exhibit greater cognitive benefit. Motor symptom severity was quantified using postoperative UPDRS Part III scores in the DOPA-ON, ON-DBS condition (see Table 1). However, due to substantial missing data, we were limited to this single motor condition for analysis. To assess whether the performance in the mental rotation task correlated with the motor capabilities, we computed a Spearman’s correlation between error rates during ON-DBS (averaged across rotation angles and items, reflecting the main effect of stimulation) and UPDRS scores (DOPA-ON, ON-DBS) across all subjects. The analysis revealed no significant correlation (*p* > 0.05), suggesting that individual cognitive performance under DBS was not directly associated with the degree of motor function.

### Limbic STN stimulation was negatively associated with performance in mental rotation task

**Fig. 3:**
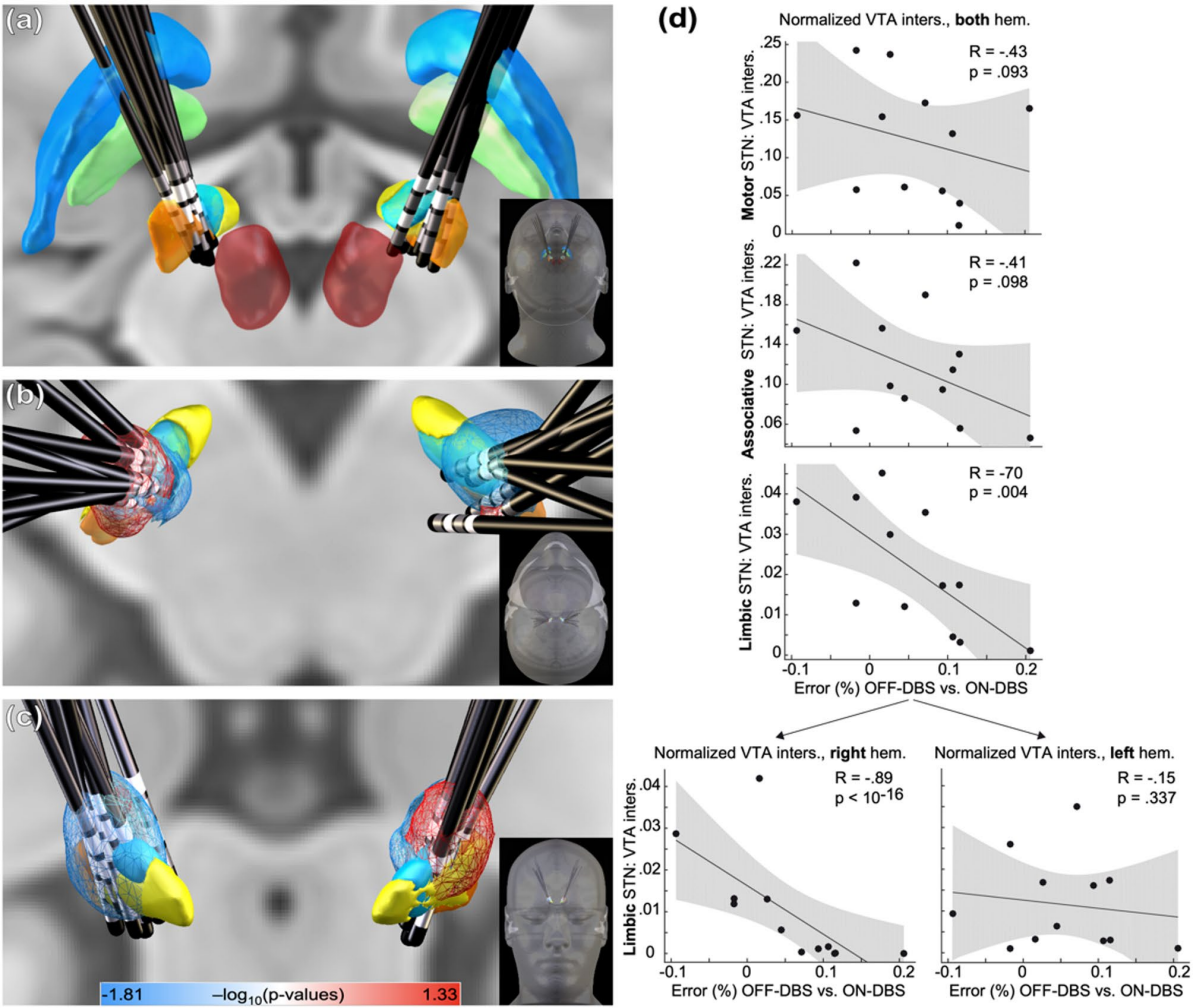
Relationship between performance in mental rotation task and electrode placement. (**a**) 3D reconstruction of DBS leads from PD patients overlaid on the DISTAL atlas^55^, posterior view. Anatomical structures are color-coded as follows: globus pallidus pars externa (blue), globus pallidus pars interna (green), red nucleus (red), and STN sub-regions—motor (orange), associative (cyan), and limbic (yellow). (**b**–**c**) Voxelwise maps showing significant correlations between stimulation location and error rate (p-maps from two-tailed Wilcoxon signed-rank tests), overlaid on the STN sub-regions. Dorsal (**b**) and frontal (**c**) views are shown. (**d**) Correlation between the change in error rate (OFF-DBS minus ON-DBS) and electrode placement within the STN motor, associative, and limbic sub-regions, as defined by the DISTAL atlas. Given the significant correlation observed in the limbic sub-region, a post hoc analysis was conducted to differentiate effects between the left and right STN. The grey shading indicates 95% confidence intervals. Regression line and individual subject data points are displayed. VTA: volume of tissue activated; inters.: intersection; hem.: hemisphere.

We anticipated that the anatomical location of stimulation within the STN would influence performance in the mental rotation task. To identify regions where stimulation was associated with above-mean (“sweet spots”) or below-mean (“sour spots”) error rates, we used VTA-based probabilistic stimulation mapping in MNI space. To assess whether stimulation location was associated with performance changes, we performed Spearman’s correlations between the change in error rate (OFF-DBS minus ON-DBS) and the VTA within bilateral motor, associative, and limbic subregions of the STN. A significant negative correlation was observed for the limbic STN (*r* = -0.7, *p* = 0.004, Fig. 3d), indicating that a greater VTA in this subregion was associated with increased error rates. Correlations for the motor and associative subregions were not significant (*p* > 0.05; to account for multiple comparisons, the Bonferroni-adjusted alpha level for the three correlations is p < 0.017).

To differentiate the effect between hemispheres, we conducted separate follow up Spearman’s correlations for the left and right limbic STN. A significant negative correlation was observed in the right hemisphere (*r* = − 0.89, *p* < 0.001), but not in the left hemisphere (*r* = − 0.15, *p* = 0.337, Fig. 3d bottom), suggesting that increased stimulation of the right limbic STN was specifically associated with impaired cognitive performance.

The robustness of the probabilistic stimulation mapping was assessed via permutation testing (1000 permutations, “leave-nothing-out” approach), revealing a strong correlation between voxelwise stimulation and task performance (*r* = 0.85, *p*(perm) = 0.036, Fig. 3b, c). Model generalizability was further supported by a tenfold randomized cross-validation (*r* = 0.56, *p* = 0.038, Fig. 3b, c), indicating that the location of stimulation in limbic STN, specifically in the right hemisphere, plays a critical role in modulating cognitive performance in the mental rotation task. Given the small sample size, this analysis should be interpreted cautiously and is considered exploratory.

As a sensitivity analysis, leave-one-out cross-validation (LOOCV) was performed, resulting in a lower and non-significant correlation (r = 0.24, *p* = 0.2). This variability across validation approaches indicates limited stability of predictive estimates in this sample.

### Response times were consistent across DBS conditions and items

To assess the effect of DBS on response times, a 4 × 2 × 2 ANOVA was conducted with the within-subject factors *rotation* (0°, 90°, 180°, 270°), *view* (mirrored, identical), and *stimulation* (ON-DBS, OFF-DBS), and response times (ms) as the dependent variable. Results revealed a significant main effect of *rotation* (F_3,33_ = 4.550, *p* = 0.009, η^2^_p_ = 0.293), suggesting that performance differed across rotation angles. Follow-up t-tests were significant for the comparison between rotation angles 0° vs 180°, indicating that response times were fastest in 0° (mean time: 8497 ± 242 ms, range 8138–9007 ms) and longest in 180° (mean time: 8706 ± 293 ms, range 8321–9300 ms), while all other comparisons between rotation angles were not significant. No other main effects or interactions were significant (all *p*-values > 0.05).

**Fig. 4:**
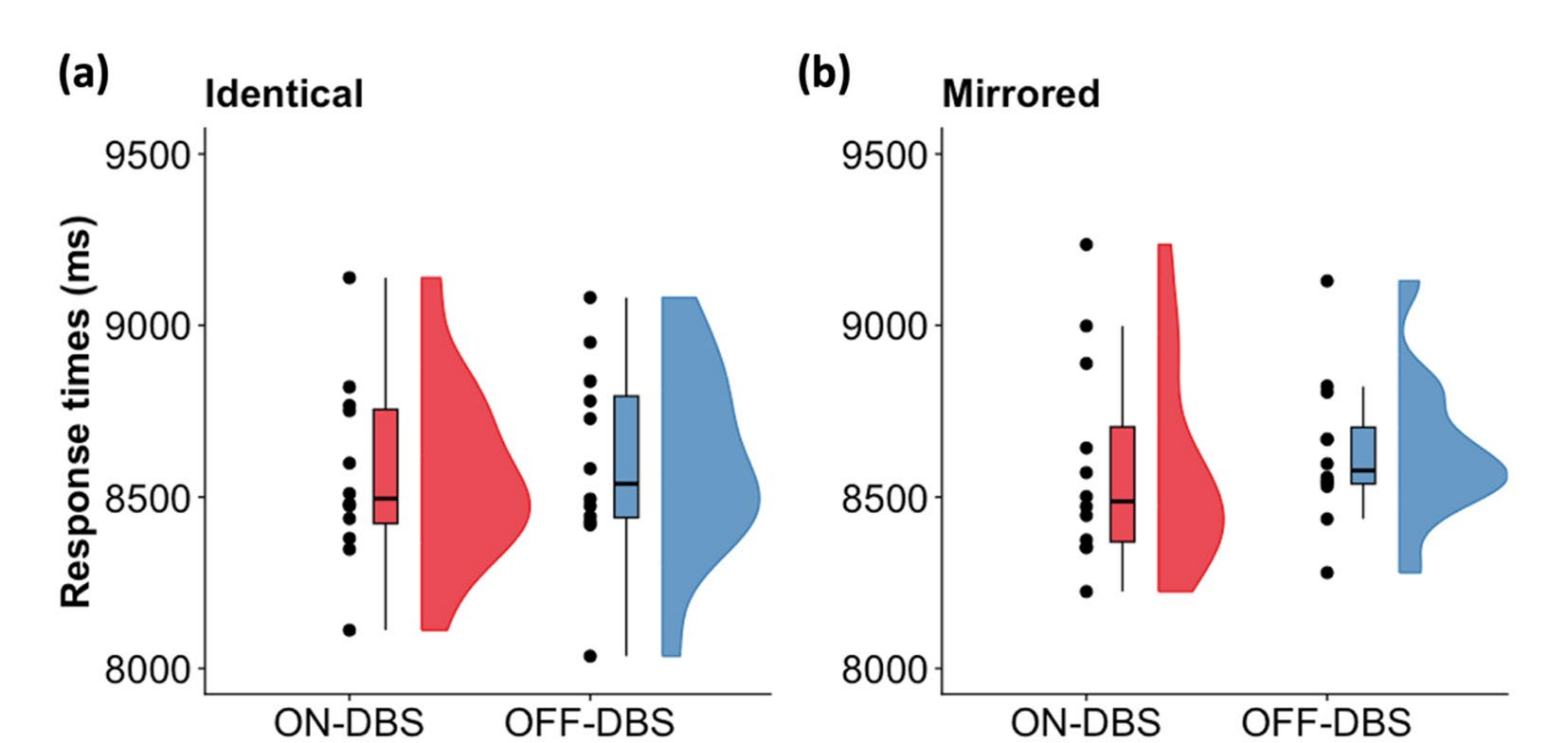
Response times were comparable across DBS conditions and items. (**a**) Comparison of response times for identical items with ON-DBS (red) and OFF-DBS (blue) indicated comparable performance. Performance was averaged over all rotation angles (0°, 90°, 180°, 270°). Single subject data, boxplots, and distributions are shown. (**b**) Comparison of response times for mirrored items with ON-DBS (red) and OFF-DBS (blue) indicated comparable performance. Performance was averaged over all rotation angles (0°, 90°, 180°, 270°). Single subject data, boxplots, and distributions across are shown. Note: To avoid response time biases, patients were instructed to withhold their response until the auditory cue signalled the end of the 8-s stimulus presentation and to respond immediately thereafter.

In the study, a fixed presentation window of 8 s was used to avoid any inter-interval differences in response times across patients. To examine whether response times were consistent, we additionally conducted an analysis averaging across rotation levels to increase statistical power for detecting potential DBS-related effects. We performed a 2 × 2 ANOVA with within-subject factors *view* (identical, mirrored) and *stimulation* (ON-DBS, OFF-DBS) with response time as dependent variable. This analysis revealed no significant main effects or interactions (all *p*-values > 0.05, Fig. 4), suggesting that response times were comparable both DBS conditions and identical and mirrored items. To follow up these effects and assess if the data favoured the null hypothesis compared to the alternative hypothesis^38^, we performed the same analysis using Bayesian ANOVA. Results revealed substantial evidence (BF_exclusion_ = 5.993) for the null hypothesis (i.e., there is no difference between response times) compared to the alternative hypothesis (i.e., there is a difference between response times). This indicated comparable response times for patients with and without DBS and items (identical and mirrored), as expected, given the fixed-response paradigm of this experiment.

### Performance changes in accuracy and response times did not correlate

To investigate whether our results were mediated by a trade-off between changes in accuracy and response times (i.e., speed-accuracy trade-off), we conducted a Spearman’s correlation between the change in error rate (OFF-DBS minus ON-DBS) and the change in response times (OFF-DBS minus ON-DBS). Results were not significant (*r* = − 0.189, *p* = 0.558, *CI* = [− 0.688, 0.432]), suggesting that we did not observe a speed accuracy trade-off for our data.

## Discussion

In this study, we investigated the modulatory effects of STN-DBS on mental rotation in PD. In line with our hypothesis, we found that STN-DBS significantly improved task performance, particularly for stimuli rotated by 180° in the identical condition. Additionally, we observed that stimulation within limbic regions of the right STN was negatively correlated with performance, suggesting that stimulation site within the STN plays a critical role in modulating cognitive outcomes. These findings provide novel insights into the cognitive effects of DBS in PD and contribute to a growing body of evidence indicating that, while DBS is primarily a motor intervention, it also has measurable effects on higher-order cognitive processes.

### DBS improved performance in mental rotation

In line with our hypothesis, we found that DBS improved performance on the mental rotation task, as indicated by fewer errors in the ON-DBS compared to OFF-DBS condition. Previous studies have shown that PD patients often exhibit impairments in mental rotation^8–10,27^, indicative of a more fundamental cognitive deficit (i.e., visuospatial functioning) caused by the disease. Our findings extend these observations by demonstrating that STN-DBS was associated with improved performance in PD patients’ mental rotation abilities in our paradigm. Importantly, these improvements in accuracy were not accompanied by longer response times, suggesting a genuine enhancement in performance rather than a change in response strategy. The DBS effect was most pronounced for stimuli rotated at 180°, which typically place the highest demands on cognitive processing. It is well-established that performance in mental rotation tasks worsens with increasing angular disparity due to task difficulty^25,59^. While these previous studies have primarily focused on response times^25,59^, our findings indicate that this pattern also applies to error rates.

While DBS is widely recognized for its efficacy in treating motor symptoms of PD, our results suggest that it also has a beneficial effect on cognitive functions such as mental rotation. This is in line with findings indicating beneficial effect of STN-DBS in visuospatial attention^14,23^. Beyond improvements in visuospatial functions, STN-DBS has been shown to enhance other cognitive abilities such as executive processes, working memory, and cognitive flexibility as well^12,13^. For instance, STN-DBS significantly improved temporal prediction performance in PD patients to a level comparable to healthy controls^32^. These cognitive benefits are believed to stem from DBS-induced modulation of pathological neural activity in PD^60,61^. Our findings align with previous reports showing DBS-related changes in cognitive functioning^12–14,23^, and provide evidence for beneficial effect of STN-DBS on cognitive performance.

It should be noted, however, that all patients in the present study were stimulated using conventional high-frequency STN-DBS. Therefore, our conclusions are restricted to this stimulation regime and cannot be generalized to alternative frequency settings. Recent studies suggest that frequency-specific modulation of cognitive processes may occur, particularly in the theta range. For example, theta-frequency stimulation of the STN has been shown to improve working memory performance in patients with Parkinson’s disease^62^, and to enhance conflict resolution through modulation of frontal networks^63^. These findings indicate that stimulation frequency may differentially engage cognitive networks, potentially via mechanisms not captured by spatial VTA-based modelling. While high-frequency stimulation remains the clinical standard, future studies should systematically investigate how frequency-specific stimulation patterns interact with stimulation location to modulate cognitive function.

### Lateralisation of STN in mental rotation

In our study, we found that bilateral STN-DBS improved performance on the mental rotation task. We also observed that electrode placement within the right STN (in limbic regions) negatively correlated with task performance, suggesting that stimulation site within the STN plays a critical role in modulating cognitive outcomes. These findings provide further evidence that the STN, and by extension the basal ganglia, might contribute to higher-order cognitive processes such as mental rotation. Moreover, our results might support the notion that basal ganglia functions are lateralised, as previously proposed^16,22^. The basal ganglia are considered to be a key component of a broader cortico-subcortical network associated with mental rotation^29,64,65^. Since the basal ganglia receive extensive projections from the cortex^66^, they are ideally positioned to integrate visuospatial information and to facilitate the selection and maintenance of neural activity required for mental rotation^64^. Converging evidence from lesion and stimulation studies indicates that in particular the right-sided basal ganglia play a dominant role in these processes^14,23,64^. A case study by Harris et al. (2002) described a patient with a right basal ganglia lesion (affecting the caudate nucleus, putamen, and globus pallidus) who exhibited severe impairments in mental rotation. This patient had a profound deficit in mentally transforming objects, particularly when attempting to map an object’s reference frame to their own egocentric perspective, while their basic visuospatial and object recognition abilities remained largely intact^64^. Further support for the functional lateralisation of the basal ganglia stems from STN-DBS studies demonstrating distinct effects of left versus right stimulation. Both Witt et al., (2006) and Schmalbach et al. (2014) reported that STN-DBS modulates visuospatial attention depending on laterality. Specifically, PD patients with left STN stimulation (i.e., right STN-DBS off) showed reduced visuospatial attention toward the contralateral visual field, which was restored when right STN stimulation was activated^14,23^. It is therefore plausible that disturbances or overstimulation within the networks linked to the right STN may compromise these functions, which could account for the results observed in our study. At the same time, the improvements in mental rotation performance observed could also be interpreted as reflecting more efficient basal ganglia functioning under STN-DBS. In this view, the observed findings may indicate a reduced cognitive load on executive functions that are otherwise recruited to compensate for basal ganglia-related inefficiency. Taken together, these findings underscore the functional lateralisation of the basal ganglia and suggest that the right basal ganglia network, including the STN, may play a role in higher-order visuospatial cognition such as mental rotation. Our results might extend these findings by showing that potential (over)stimulation of the right STN, particularly in its limbic subregion, was associated with worsened performance, while we did not find any association between the left STN and performance. While the observed association between right limbic STN stimulation and impaired performance was pronounced, it should be interpreted with caution given the small sample size. Visual inspection of the data does not suggest that the effect is driven by outliers; however, variability across validation approaches indicates that the stability of this lateralized finding is limited and should therefore be interpreted cautiously.

### Topography of STN stimulation and its functional consequences

Electrode placement within the STN plays a critical role in modulating cognitive outcomes. In line with our hypothesis, we anticipated that location of stimulation would influence task performance with more ventral placement of the DBS electrode (i.e., closer to the substantia nigra and limbic areas) leading to more errors, and more dorsal placement leading to fewer errors. Our results supported the first part of this hypothesis, showing that more ventral stimulation sites were indeed linked to impaired performance. However, we did not find evidence that more dorsal stimulation was associated with enhanced cognitive accuracy.

Dorsal STN placement of the DBS electrode is often associated with more favourable motor outcomes and is widely considered the clinically optimal stimulation target for alleviating PD motor symptoms. For example, Zhang et al. (2021) demonstrated that the closer the electrode placement was to the dorsolateral sensorimotor region of the STN, the greater the improvement in motor symptoms. Compared to lateral placement, ventral, medial or anterior placement especially in the left STN are associated with deteriorated cognitive symptoms such as worsened executive functions, verbal memory^15,19,20,67^, and verbal fluency^68^. The stimulation of limbic STN subregions has been associated to other detrimental effects, including the emergence of manic symptoms in PD patients^21^. In this context, our findings further emphasize that stimulating the limbic STN subregions not only poses neuropsychiatric risks but potentially also lead to deficits in cognitive performance, specifically in mental rotation. This might have wide ranging consequences for daily life functioning and rehabilitation for patients, as the role of visuospatial information processing for PD symptoms has already been shown^69^. Thus, our results support the notion that the precise anatomical targeting of DBS electrodes, and the networks they engage, plays a critical role in shaping both cognitive and motor-cognitive outcomes.

### Limitations

Several limitations should be acknowledged. First, the relatively small sample size limits statistical power and generalizability. However, this constraint primarily reflects practical challenges such as patient availability and clinical feasibility. Second, UPDRS data were incomplete, limiting our ability to correlate cognitive and motor outcomes robustly. The values were missing randomly and not due to specific clinical characteristics but rather attributable to clinical feasibility and logistical and practical factors. Third, the lack of eye-tracking data limits the possibility to rule out visual scanning differences as a contributing factor (see for a review^70^). Specifically, with STN-DBS turned off, PD patients have been reported to express gaze impairments such as shorter saccades and rightward viewing bias which improved with STN-DBS^71^. Although it is unlikely that these effects fully explain our findings, they might have influenced patients’ visual scanning performance necessary for the performance in the mental rotation task. Future studies should include eye-tracking to directly assess oculomotor behaviour in relation to DBS and cognitive task performance. Fourth, although we employed established methods for electrodes localization and stimulation modelling, the results must be interpreted with caution. As noted by Dembek (2022), even advanced approaches face limitations in accurately localizing the neuroanatomical origins of DBS effects. Limitations include imaging inaccuracies, lead localization, and assumptions within stimulation models, particularly binarized VTAs, which can affect the size and shape of identified sweet or sour spots. Fifth, the stability of predictive estimates should be interpreted with caution given the small sample size. Differences across validation approaches (k-fold vs. LOOCV) highlight the variability of model performance estimates and underline the exploratory nature of these analyses. Sixth, stimulation frequency was not explicitly modelled, as the VTA approach implemented in Lead-DBS does not incorporate frequency as a parameter. Although stimulation frequencies in our cohort were largely homogeneous and within the clinically typical high-frequency range, potential effects of alternative stimulation regimes (e.g., low-frequency or patterned stimulation) on cognitive performance cannot be excluded and should be addressed in future studies. Finally, the observed lateralized effect in the right limbic STN should be interpreted cautiously, as its stability is limited in this small sample and cannot fully exclude influence from individual observations. While local DBS mapping remains a powerful tool, its outcomes must therefore be interpreted in light of these methodological constraints.

### Conclusion

Our findings show that STN-DBS can enhance cognitive performance in PD patients during a mental rotation task, pointing to its effects beyond motor symptom control and to the involvement of the basal ganglia in higher-order visuospatial processing. At the same time, these results could also reflect more efficient basal ganglia functioning under STN-DBS, releasing the cognitive load on executive functions otherwise recruited to compensate for basal ganglia-related inefficiency. Stimulation of non-motor regions was linked to impaired performance, emphasizing the need for individualized targeting. Together, these results highlight the importance of considering cognitive outcomes in optimizing DBS treatment and encourage further research into the cognitive consequences of subcortical neuromodulation in PD.

## Data Availability

The datasets generated for this study and the analysis codes are available on request to the corresponding author AG.
